# Language dominance and order of acquisition affect auditory translation priming in heritage speakers

**DOI:** 10.1177/17470218221091753

**Published:** 2022-05-07

**Authors:** Rachel Soo, Philip J Monahan

**Affiliations:** 1 Department of Linguistics, University of British Columbia, Vancouver, British Columbia, Canada; 2 Department of Language Studies, University of Toronto Scarborough, Toronto, Ontario, Canada; 3 Department of Linguistics, University of Toronto, Toronto, Ontario, Canada; 4 Department of Psychology, University of Toronto Scarborough, Toronto, Ontario, Canada

**Keywords:** Language dominance, heritage speakers, lexical access, bilingualism, psycholinguistics

## Abstract

Late second language (L2) learners show translation priming from the first language (L1) to the second language (L1–L2), while L2–L1 effects are inconsistent. Late L2 learners also acquire the L2 after the L1 and are typically less dominant in the L2. As such, the relative contribution of language dominance and order of acquisition is confounded in these results. Here, Cantonese heritage and native speakers are tested in an auditory translation priming paradigm. As heritage speakers first learn Cantonese (L1) but later become dominant in English (L2), this profile allows for the potential dissociation of dominance and order of acquisition in translation priming. If order of acquisition is the primary factor, stronger priming is expected in the L1–L2 (Cantonese–English) direction; however, if dominance plays a stronger role, priming is expected in the L2–L1 (English–Cantonese) direction. Native speakers showed stronger L1–L2 priming, consistent with previous findings, while heritage speakers showed priming in both directions, and marginally larger L2–L1 priming. Treating language dominance as a continuous variable revealed that L1–L2 priming correlated with increased Cantonese dominance, while L2–L1 priming marginally correlated with increased English dominance. Collectively, these results suggest that both language dominance and order of acquisition help explain translation priming findings and bilingual lexical processing, generally. Overall, they invite a rethinking of the role of both variables in bilingual lexical access for speakers with different language dominance profiles.

## Introduction

Hearing a word spoken in one language often activates similar words in the other language in bilingual speakers (Ju & Luce, 2004; Spivey & Marian, 1999). Proficiency in the first (L1) and second (L2) languages, however, affects this inter-lexicon activation (Kroll & Stewart, 1994; Kroll & Tokowicz, 2005; Lee-Ellis, 2012; Van Hell & De Groot, 2008). A common method to investigate cross-linguistic activation is translation priming, although much of this work has focused on late L2 learners (Altarriba & Basnight-Brown, 2007). In translation priming paradigms, the prime is a lexical item from one language, and the target is its translation equivalent in the other language. Robust translation priming is observed from the L1 to the L2 (Altarriba, 1992; Chen & Ng, 1989; Frenck & Pynte, 1987; Jin, 1990; Keatley et al., 1994; Mott et al., 2020; Schwanenflugel & Rey, 1986; Smith et al., 2019), while translation priming effects from the L2 to the L1 are inconsistent (Altarriba, 1992; Grainger & Frenck-Mestre, 1998; Jiang, 1999; Jiang & Forster, 2001; McPhedran & Lupker, 2021; Meade et al., 2018; Nahrkhalaji et al., 2014; Smith et al., 2019; Woutersen et al., 1994).

Two variables that characterise bilinguals are conflated in most late L2 learners: order of acquisition and relative language dominance (Birdsong, 2014). Specifically, late L2 learners acquire their L1 first and are typically more dominant in their L1 relative to their L2. Thus, it is unclear whether the lack of robust L2–L1 priming is due to the L2 being learned later or due to the L2 being less dominant. Studies using translation priming with early L2 learners provide some insight. Gollan et al. (1997) observed only robust L1–L2 priming with highly proficient Hebrew–English bilinguals, and Dudsic (1999) found similar results in highly proficient Mandarin–English bilinguals. Because asymmetric priming effects are observed, and there are unlikely to be large language dominance differences for highly proficient bilinguals, these results suggest that dominance may not play a role in translation priming asymmetries. On the contrary, Duñabeitia et al. (2010) observed symmetric translation priming in balanced Basque–Spanish bilinguals, suggesting that language dominance may indeed drive translation priming. At the same time, it is unclear to what extent these conflicting results are driven by differences in orthographic scripts across languages (Frost, 2012; Katz & Frost, 1992), as Mandarin and Hebrew utilise distinct orthographic scripts from English, while Basque and Spanish utilise the same script.

To examine the relative contributions of order of acquisition and language dominance, we tested a bilingual population, namely, heritage speakers, for whom their L2 is more dominant despite being acquired after the L1. Heritage speakers are often raised with an L1 that represents a (local) minority language at home but later became more dominant in the language of the wider community (Benmamoun et al., 2013; Montrul, 2012). Although both heritage speakers and late L2 learners acquire the L2 after the L1, heritage speakers are more dominant in the L2, while late L2 learners remain dominant in the L1. This linguistic profile makes it possible to examine the relative contributions of order of acquisition and language dominance more closely in translation priming. In the only test of translation priming with heritage speakers to the best of our knowledge, Lee-Ellis (2012) found larger L1–L2 (Korean–English) priming in Korean native speakers and larger L2–L1 (English–Korean) priming in Korean heritage speakers. These results suggest that language dominance, as opposed to order of acquisition, drives translation priming effects in bilinguals: Both groups acquired Korean first, but native speakers were dominant in Korean, while heritage speakers were dominant in English.

In nonselective models of bilingual lexical processing (e.g., Multilink; Dijkstra et al., 2019), lexical items in the L1 and L2 are stored in an integrated lexicon. Differential patterns of language usage conspire to produce different resting activation levels for lexical items from the L1 and the L2, and these resting activation level differences account for translation priming asymmetries (Smith et al., 2019). This contrasts with earlier selective models of bilingual lexical access. Selective models accounted for priming asymmetries with two distinct, but related, aspects of the bilingual lexicon: concept mediation and lexical association (Potter et al., 1984). In the Revised Hierarchical Model (Kroll & Stewart, 1994), order of acquisition is the relevant bilingual characteristic: Lexical items in the L1 and L2 are mapped to the same conceptual representation in the lexicon. Robust L1–L2 priming effects result from a stronger link between the L1 lexical item and the concept, while the less robust and inconsistent L2–L1 priming is likely due to the comparatively weaker link between the L2 lexical item and the same concept (Jiang & Forster, 2001; Lee-Ellis, 2012).

Nonetheless, much of the translation priming literature has focused on the visual modality (Altarriba & Basnight-Brown, 2007). Consequently, the bilingual lexical activation models that have either been proposed to account for these data or modified to account for these data are principally based on visual word recognition (Dijkstra et al., 2019; Dijkstra & van Heuven, 2002; Kroll & Stewart, 1994); their application to auditory word recognition is less obvious. In addition to a general lack of auditory translation priming experiments, visual translation priming paradigms are not always viable, as literacy cannot be assumed in all bilingual populations. In the only auditory translation priming study of which we are aware, Szakay et al. (2016) observed robust L1–L2 priming, and L2–L1 priming was found only when the L2 was Māori and the L1 was Māori–English, two languages that index Māori ethnic identity. Moreover, previous results with highly proficient bilinguals still observe asymmetric priming effects (Dudsic, 1999; Gollan et al., 1997), which suggests that dominance might not be the primary factor in translation priming, contrary to previous work with heritage speakers (Lee-Ellis, 2012). Understanding the interaction of these factors is important in the context of bilingual lexical processing models, where a myriad of such bilingual factors may account for priming asymmetries. With this in mind, we test Cantonese heritage and native speakers in an auditory translation priming paradigm. Our sample of heritage speakers first learned Cantonese (L1) and later became dominant in English (L2). Participants were auditorily presented with prime and target translation equivalents and made a lexical decision to the target. Both the L1–L2 (Cantonese–English) and L2–L1 (English–Cantonese) priming directions were tested. For native Cantonese speakers, we predicted L1–L2 priming and little to no L2–L1 priming, consistent with the previous literature (see Altarriba & Basnight-Brown, 2007). For heritage speakers, if order of acquisition drives the direction of translation priming effects, we predict stronger L1–L2 priming. If, however, language dominance drives the direction of translation priming effects, we predict stronger L2–L1 priming (Lee-Ellis, 2012).

## Method

### Participants

Cantonese–English bilinguals (*n* = 171) were recruited both from the University of Toronto and Prolific (https://prolific.co/) and completed a web-based auditory translation priming lexical decision task. Participants reported no known linguistic, hearing, or neurological deficits and provided written informed consent prior to the experiment. Participants were compensated for their time either monetarily or with course credit. The experiment was approved by the Office of Research Ethics at the University of Toronto. Twenty-two participants who did not speak Cantonese were removed from further analysis. Thirty-nine participants were removed from the analysis for failing to achieve an accuracy rate greater than 75% on the lexical decision task. In total, 108 participants were included in the analysis (mean age = 23.6 years, *SD* = 5.0 years, 66 females).

**Figure 1.**
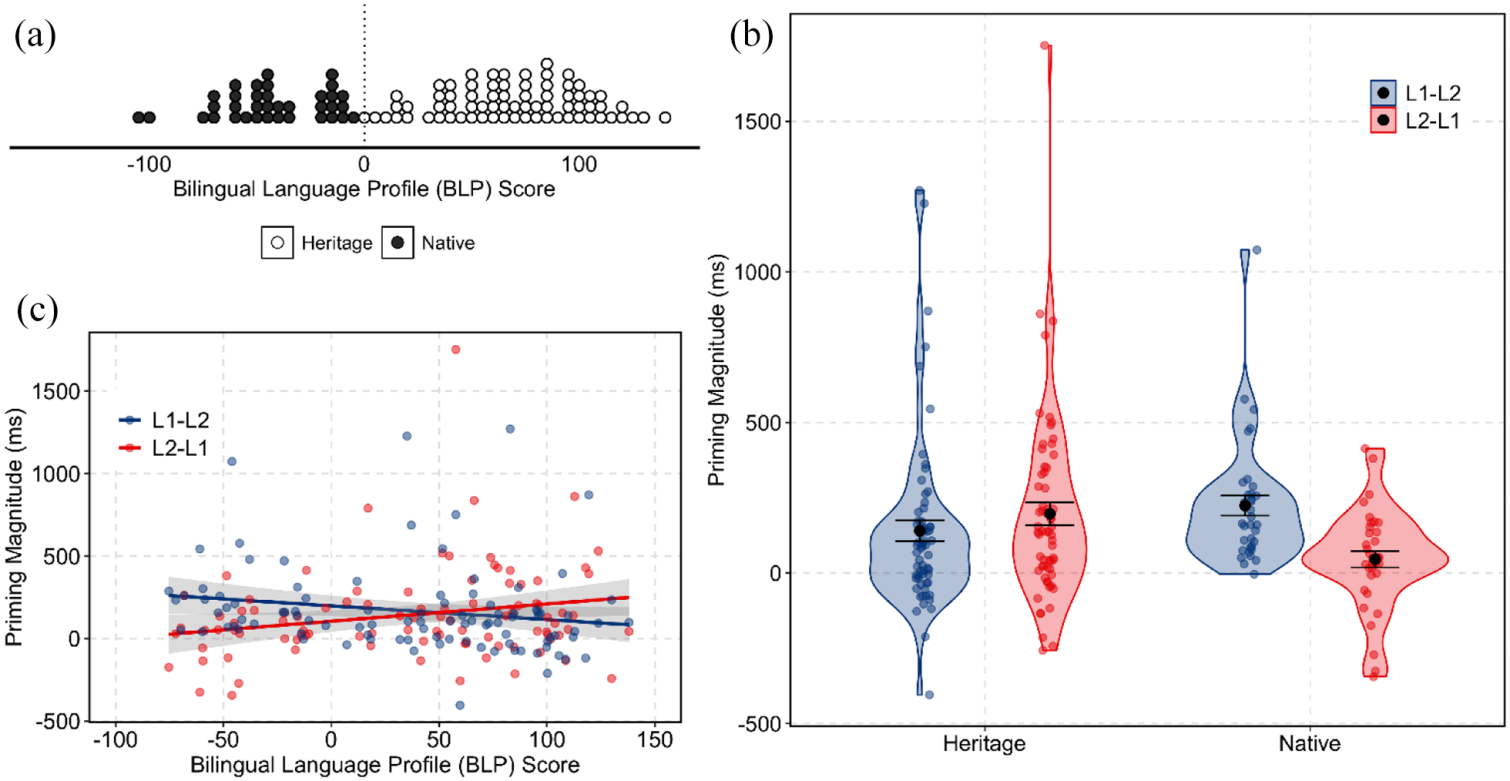
(a) Distribution of language dominance scores as measured with the Bilingual Language Profile (BLP; Gertken et al., 2014) for heritage and native Cantonese speakers included in our sample. (b) Priming magnitude density distributions (reaction times for related trials subtracted from unrelated trials on a by-participant basis) in the auditory translation priming experiment. A positive priming magnitude indicates that the related items were responded to more quickly compared with the unrelated items. The aggregate mean and standard error of the mean are also presented. Points represent individual participant priming magnitudes for each translation direction (L1–L2: Cantonese–English, blue; L2–L1: English–Cantonese, red). (c) Correlation of priming magnitudes (ms) with BLP dominance scores in each translation direction (L1–L2: Cantonese–English, blue; L2–L1: English–Cantonese, red).

All participants were born to Cantonese-speaking parents and learned Cantonese first (mean age of acquisition = 1.6 years, *SD* = 1.1 years), followed by English (mean age of acquisition = 4.4 years, *SD* = 2.2 years). Participants were divided into two groups: Cantonese heritage and “native speakers” (see Cheng et al., 2021, for a discussion of the term “native speaker”), based on their “dominance score” obtained from the Bilingual Language Profile (BLP; Gertken et al., 2014) questionnaire. The BLP questionnaire surveys language history, attitudes, background, and usage in various social contexts. Many of these factors are used to define whether a given bilingual is a heritage speaker (Benmamoun et al., 2013; Chang & Yao, in press). Responses are then converted into a quantified composite measure called a “dominance score” on an arbitrary –218 to 218 scale. Here, BLP scores greater than 0 reflect English dominance relative to Cantonese, while BLP scores less than zero reflect greater Cantonese dominance relative to English. Participants were categorised as heritage speakers if their BLP score was greater than zero (*n* = 72) and as native speakers if their BLP score was less than zero (*n* = 36). Figure 1a presents the distribution of BLP dominance scores for all participants. In addition to the BLP, participants also completed a separate language background questionnaire. Using a 7-point Likert-type scale, participants self-reported their English and Cantonese speaking and understanding proficiency. Overall, heritage speakers self-reported higher speaking and understanding abilities in English than in Cantonese (Cantonese: median speaking = 5.0, median understanding = 5.6; English: median speaking = 6.8, median understanding = 6.8), while native speakers self-reported higher speaking and understanding abilities in Cantonese than in English (Cantonese: median speaking = 6.9, median understanding = 6.9; English: median speaking = 5.5, median understanding = 5.8). Full participant details are provided in the Supplementary Material.

### Stimuli

Sixty real-word Cantonese–English related translation prime-target pairs were selected (e.g., *ci3so2* “toilet”—*toilet*). All Cantonese words were disyllabic. English words varied between one and three syllables (mean syllable length = 1.8, *SD* = 0.37; mean phoneme length = 5.1, *SD* = 1.2). The prime and target in each pair were always noncognates in distinct languages. In addition to these related translation pairs, each prime–target pair was matched with an unrelated, real-word Cantonese prime that was not a translation equivalent (e.g., *lai5mat6* “present”—*toilet*). Finally, these Cantonese–English pairs were matched with the corresponding English–Cantonese related and unrelated pairs (e.g., *toilet*—*ci3so2* “toilet” and *present*—*ci3so2* “toilet,” respectively). This produced translation priming pairs in the Cantonese–English direction (i.e., L1–L2) and in the English–Cantonese direction (i.e., L2–L1 priming). Table 1 provides an example stimulus set with the factors priming direction (L1–L2/L2–L1) and translation relatedness (Related/Unrelated). In total, 240 unique real-word prime–target pairs were created and distributed across eight counterbalanced lists in a Latin Square design. Priming direction was consistent in each list: Four lists contained Cantonese primes and English targets, while the other four lists contained English primes and Cantonese targets. In the end, each of the eight lists contained 30 experimental trials, 15 related prime–target pairs and 15 unrelated prime–target pairs.

**Table 1.** Example stimuli set from the translation priming experiment.

Direction	Language	Relatedness	Prime	Target
L1–L2	Cantonese–English	Related	廁所 ci3so2 “toilet”	Toilet
L1–L2	Cantonese–English	Unrelated	禮物 lai5mat6 “a present”	Toilet
L2–L1	English–Cantonese	Related	Toilet	廁所 ci3so2 “toilet”
L2–L1	English–Cantonese	Unrelated	A present	廁所 ci3so2 “toilet”

An additional 60 word–nonword prime–target pairs were created. The Cantonese nonwords were phonotactically legal disyllabic nonwords that were confirmed to be highly unfamiliar to Cantonese–English bilinguals (Chan et al., 2020). All English nonwords were disyllabic, pronounceable, matched in length with the real English words (mean phoneme length = 5.7, *SD* = 1.0), and obtained from the English Lexicon Project (Balota et al., 2007). Thirty pairs contained Cantonese primes and English nonword targets (i.e., L1–L2 priming), while the other 30 pairs contained English primes and Cantonese nonword targets (i.e., L2–L1 priming). These word–nonword pairs were included in each list according to the appropriate priming direction. In total, each list contained 30 real-word targets and 30 nonword targets, producing a total of 60 trials per list. Finally, all stimuli were recorded by a phonetically trained Cantonese–English bilingual. Acoustic recordings were sampled at 44.1 kHz with 16-bit depth in Praat (Boersma & Weenink, 2020). Stimuli were mean intensity normalised to 65 dB SPL.

#### Translation equivalent pretest

Following Jiang (1999), the related pairs were submitted to a pretest. This was done to ensure that the Cantonese and English translations in each of the related pairs were unique. Three Cantonese–English bilinguals provided Cantonese translations for the English words, and three different Cantonese–English bilinguals provided English translations for the Cantonese words. Only items for which there was agreement among 4/6 of the speakers across both priming directions were included in the experiment (mean inter-rater agreement = 5.3/6 participants).

#### Familiarity rating pretest

As our target population is heritage speakers, who are more dominant in English, it was important to ensure that all Cantonese items were familiar to our target sample; however, a suitable heritage speaker corpus that contained our experimental items was unavailable, making it difficult to control for lexical frequency. To this end, we recruited 16 heritage speakers (mean BLP = 81.1, *SD* = 32.0) and 8 native speakers (mean BLP = –42.2, *SD* = 25.3) who did not participate in the main experiment to complete a familiarity rating task. Both the Cantonese and English target words were included, and participants were randomly assigned to rate either the English words or the Cantonese words. Following the familiarity rating procedure in Lee-Ellis (2012), on each trial, participants both listened to and saw each word visually presented on their computer monitor. English items were accompanied only by their English orthographic form, while Cantonese items were presented in both Chinese characters and Jyutping (i.e., Romanized Cantonese), as Cantonese literacy cannot be assumed in the heritage speaker population. Participants assigned a familiarity rating to each word on a 7-point Likert-type scale, with 1 representing an “unfamiliar” word and 7 representing a “familiar” word. Each participant rated all 120 experimental items in the language to which they were assigned. The median rating for all items was 7 (and all means were above 5.5/7), suggesting that all words were familiar to our target population.

### Procedure

The experiment was delivered online using Gorilla (Anwyl-Irvine et al., 2020). After providing informed consent, participants took part in a short listening task to establish that they were wearing adequate headphones (Woods et al., 2017). Participants that failed to achieve 80% accuracy on the task were not permitted to proceed.

As mentioned, each pair in a stimuli quadruple (see Table 1) varied by translation relatedness and direction of priming, and was placed in one of eight distinct experimental lists in a Latin square design. Four of these lists tested priming in the L1–L2 direction, while the other four tested priming in the L2–L1 direction. The experiment was a within-subjects design in which participants were randomly assigned to two lists presented as separate blocks with a break in between. In one block, participants heard Cantonese primes preceding English targets (i.e., L1–L2 priming). In the other block, participants heard English primes preceding Cantonese targets (i.e., L2–L1 priming). Block order was counterbalanced across participants. No participant heard multiple pairs from the same quadruple. Across the entire experiment, there were a total of 120 trials: 60 trials in the first block with targets in one language, 60 trials in the second block with targets in the other language.

At the start of the first block (irrespective of the target language of each block), participants were visually presented with instructions in English, and four practice trials with English primes and Cantonese targets (L2–L1 priming). Following the practice trials, participants were presented with either an English or Cantonese version (https://www.aesoplanguagebank.com/yue.html) of *The North Wind and the Sun*. At the start of the block containing English targets, participants listened to the English version of *The North Wind and the Sun*, while at the start of the block containing Cantonese targets, they listened to the Cantonese version. The story was produced by the same speaker who recorded the experimental items. This was done to familiarise participants with the speaker’s voice and prepare them to make lexical decisions to the corresponding language of the target.

In the main task, each trial began with a fixation point (“+”) that appeared on the screen for 250 ms to indicate the start of the trial. Immediately after the fixation point, the auditory prime was presented to the participant. After a 500-ms inter-stimulus interval, the auditory target was presented to the participant, and participants made a lexical decision. Participants responded by pressing “1” on their keyboard if they heard a real word of Cantonese or English (depending on the block), or “0” if they heard a nonword of Cantonese or English. Both accuracy and reaction times (counted from the offset of the auditory target) were measured. The inter-trial interval was 1,000 ms. After the experiment was complete, participants completed the BLP questionnaire (Gertken et al., 2014) and a short language background questionnaire.

## Results

All data aggregation and visualisation were conducted using the packages {dplyr} (Wickham et al., 2020) and {ggplot2} (Wickham, 2016) in R (R Core Team, 2019). First, participants who were less than 75% accurate in the lexical decision task were excluded from the analysis (see “Participants” section). Then, trials with reaction times less than 50 ms and greater than 8 s were eliminated (2.8% of all trials). Finally, trials with reaction times ±2.5 standard deviations from an individual participant’s mean reaction time were removed (3.3% of the data). Reaction time distributions for heritage and native speakers of the Direction and Relatedness conditions are provided in the Supplementary Material. Overall, Cantonese heritage speakers were less accurate than native speakers (Heritage: *M* = 89%, *SD* = 32%; Native: *M* = 92%, *SD* = 28%); however, this is largely due to the lower accuracy for heritage speakers to Cantonese nonwords (*M* = 86%, *SD* = 34%). All other accuracy rates were above 90%. Only trials with correct responses were included in the reaction time analysis.

Reaction times were entered into a generalised linear mixed effects model with Gaussian errors (Lo & Andrews, 2015) and a log link function using the {lme4} package in R (Bates et al., 2015). The model included the simple coded fixed factors of Group (Heritage, Native), Direction (L2–L1, L1–L2), Relatedness (Related, Unrelated), and their interactions. The model’s random factor structure included random by-participant slopes for Direction, as well as random by-participant and by-item intercepts. This model was selected using stepwise model comparison based on an Akaike’s information criterion (AIC), starting from a model with random by-participant slopes for Direction and Relatedness and their interaction, as well as random by-item slopes for Direction, Relatedness, and Group and all interactions. To ensure model convergence, we used a Bound Optimization BY Quadratic Approximation (BOBYQA; Powell, 2009) optimiser. The full model output is provided in Table 2. There was a main effect of Group with native speakers (*M* = 556 ms, *SD* = 489 ms) responding faster than heritage speakers (*M* = 683 ms, *SD* = 663 ms). There was also a main effect of Direction, with participants responding to the L2–L1 direction (*M* = 607 ms, *SD* = 616 ms) faster than the L1–L2 direction (*M* = 672 ms, *SD* = 609 ms). Finally, there was a main effect of Relatedness with participants responding slower to unrelated pairs (*M* = 712 ms, *SD* = 661 ms) compared with related pairs (*M* = 578 ms, *SD* = 561 ms). In addition to these main effects, there was a Direction × Relatedness interaction and a three-way Group × Direction × Relatedness interaction. For the Direction × Relatedness interaction, we observed a larger difference between the L1–L2 and L2–L1 directions in the unrelated trials (L1–L2: 753 ms, *SD* = 687 ms; L2–L1: 668 ms, *SD* = 628 ms; Δ = 85 ms) compared with the related trials (L1–L2: 600 ms, *SD* = 520 ms; L2–L1: 555 ms, *SD* = 601 ms; Δ = 85 ms). The descriptive statistics for the three-way interaction are presented in Table 3.

**Table 2.** Output of the generalised linear mixed effects model with Gaussian errors and a log link function.

	β	*SE*	*t*	Pr(>|*t*|)	
(Intercept)	6.34	0.04	149.18	<.001	***
Direction	0.09	0.03	3.41	<.001	***
Group	–0.17	0.08	–2.02	.04	*
Relatedness	0.23	0.02	10.11	<.001	***
Direction × Group	0.08	0.05	1.80	.07	.
Direction × Relatedness	0.10	0.05	2.16	.03	*
Group × Relatedness	–0.04	0.05	–0.88	.38	
Direction × Group × Relatedness	0.35	0.09	3.82	<.001	***

Simple coding was used for all fixed factors.

Significance codes: *** represent < 0.001, ** represent < 0.01, * represents < 0.05, and . represents < 0.1.

**Table 3.** Mean reaction times in milliseconds across participants for each individual factor for the word trials only.

Group	Relatedness	Direction	
		L1–L2 (C-E)	L2–L1 (E-C)
Heritage	Related	648 (552)	576 (646)
	Unrelated	788 (742)	742 (696)
Native	Related	502 (428)	513 (487)
	Unrelated	680 (550)	540 (462)

C-E: Cantonese–English; E-C: English–Cantonese.

Standard deviations are provided in parentheses.

To determine whether priming was observed in each priming direction for each participant group, as well as whether we observed more priming in the L1–L2 direction or the L2–L1 direction, a series of post hoc comparisons were conducted using the {phia} package (Rosario-Martinez, 2015). The Holm method for multiple comparisons was used. Heritage speakers responded to the related items faster than the unrelated items (i.e., evidence for priming) in both directions, L1–L2: χ^2^(1) = 55.39, *p* < .001; L2–L1: χ^2^(1) = 72.55, *p* < .001, while native speakers showed priming only in the L1–L2 direction, χ^2^(1) = 43.04, *p* < .001, and not in the L2–L1 direction, χ^2^(1) = 1.65, *p* = .20. Priming magnitudes (Unrelated minus Related trials) by participant Group by Direction are presented in Figure 1b. For native speakers, more L1–L2 priming (Δ = 178 ms) was observed compared with L2–L1 priming, Δ = 27 ms, χ^2^(1) = 11.77, *p* < .01, while for heritage speakers, marginally more L2–L1 priming (Δ = 166 ms) was observed compared with L1–L2 priming, Δ = 140 ms, χ^2^(1) = 2.86, *p* = .09. Finally, for the two-way interaction, the L2–L1 direction resulted in faster reaction times compared with the L1–L2 direction in the unrelated trials, χ^2^(1) = 18.45, *p* < .001, but not in the related trials, χ^2^(1) = 1.28, *p* = .26.

Order of acquisition and language dominance are still conflated in the native speakers, who showed stronger L1–L2 priming than L2–L1 priming. To dissociate these factors more clearly, as well as assess relative language dominance on a more granular level, participants’ BLP dominance scores were correlated with their priming magnitudes by direction (see Figure 1c). Kendall’s rank correlation tau (Kendall, 1955) was calculated to quantify the strength and direction of the relationship between the priming magnitude and BLP dominance scores. We observed a negative correlation between language dominance and priming in the L1–L2 direction (*z* = –3.32, *p* < .001, τ = –0.23), and a marginal positive correlation between language dominance and priming in the L2–L1 direction (*z* = 1.83, *p* = .07, τ = 0.13). The more Cantonese dominant the participant was, the more L1–L2 priming was observed; the more English dominant the participant was, the more L2–L1 priming was observed.

## Discussion

Previous translation priming studies on late L2 learners have observed robust L1–L2 priming, whereas L2–L1 priming has been inconsistent and, traditionally, difficult to obtain in both visual (L2–L1; Altarriba & Basnight-Brown, 2007; Jiang, 1999; Woutersen et al., 1994) and auditory (Szakay et al., 2016) modalities; however, late L2 learners both learn the L2 later and are less dominant in the L2, making the relative contributions of order of acquisition and relative language dominance in translation priming unclear. To dissociate these factors, we tested heritage Cantonese speakers in an auditory translation priming paradigm. Our sample of heritage speakers learned the L2 (English) after the L1 (Cantonese) but crucially is more dominant in the L2. Thus, we predicted that heritage speakers would show greater L2–L1 than L1–L2 priming if dominance drives translation priming, but greater L1–L2 than L2–L1 priming if order of acquisition drives priming.

We found that heritage speakers showed marginally larger L2–L1 priming, while native speakers showed larger L1–L2 priming. Together, these results support the notion that language dominance drives translation priming, because both groups experienced stronger priming in the instances where their more dominant language acted as the prime (i.e., the L2 for heritage speakers and the L1 for native speakers). These findings are further supported on a more granular level in correlations between BLP dominance scores and priming magnitudes. Specifically, greater English dominance marginally correlated with greater L2–L1 priming, while greater Cantonese dominance correlated with greater L1–L2 priming. Although marginally more L2–L1 priming was observed compared with L1–L2 priming for heritage speakers, post hoc tests revealed that priming occurred in both directions. The same was not true for native speakers, however, for whom only L1–L2 priming was observed. The current findings are consistent with Duñabeitia et al. (2010), who observed priming in both directions for balanced Basque–Spanish bilinguals. Moreover, they are consistent with Lee-Ellis (2012), who found robust L2–L1 priming with heritage speakers. They are in contrast, however, to previous reports with highly proficient bilinguals, who failed to observe L2–L1 priming (Dudsic, 1999; Gollan et al., 1997). More broadly, these findings echo Jiang (1999) that the lack of robust L2–L1 translation priming reflects representational differences between lexical items across languages, as opposed to modality or task-specific considerations.

These results may be explained by recent models that posit nonselective access to an integrated lexicon. For example, in Multilink (Dijkstra et al., 2019), lexical representations from both languages are stored and activated in a single language node (Dijkstra & van Heuven, 2002). Priming asymmetries in unbalanced bilinguals are explained by relative L1 and L2 usage frequency (Smith et al., 2019). Less frequent use of the L2 results in lower resting activation levels. Consequently, the activation of L2 lexical items may be delayed, a phenomenon referred to as the “temporal delay assumption” (Sabourin et al., 2014). This delay is what accounts for the comparatively weaker L2–L1 priming.

More broadly, Multilink formalises language use as language proficiency; however, language proficiency results from a variety of factors. McPhedran and Lupker (2021) find support for Multilink in their masked translation priming study testing Chinese–English bilinguals, where language proficiency was defined based on self-rated reading, writing, and listening abilities. To the extent that language proficiency includes language dominance, the weaker L2–L1 priming in native speakers may be explained if the resting activation levels for the nondominant language (L2, English) are lower than the dominant language (L1, Cantonese). Moreover, language proficiency as employed by these models may ultimately encompass several bilingual factors for different bilingual groups (Chang & Yao, in press; Gathercole, 2016; Luk & Bialystok, 2013). The intersection of language dominance and order of acquisition is relevant for heritage speakers. In the current study, while heritage speakers demonstrated marginally larger L2–L1 priming compared with L1–L2 priming, we observed priming in both directions. This suggests that both order of acquisition and language dominance play a role in affecting the resting activation levels for different translation priming directions.

Understanding the interaction between different bilingual factors as they contribute to language proficiency, overall, is necessary for a holistic account of bilingual lexical processing. Previous work has focused on late L2 learners and the role of order of acquisition in translation priming (see Altarriba & Basnight-Brown, 2007, for a review); however, Lee-Ellis (2012) and the current study demonstrate that language dominance is also an important factor in heritage speaker translation priming. If models of bilingual lexical processing are to account for these empirical findings, understanding the extent to which different bilingual factors contribute to the locus of priming asymmetries is necessary. Additional work is required to investigate how lexical representations can be integrated in a lexicon considering bilingual communities with varying language dominance backgrounds.

## Conclusion

Previous translation priming studies have found robust L1–L2 priming and inconsistent L2–L1 priming; however, most of these studies test late L2 learners in the visual modality. Late L2 learner profiles conflate order of acquisition and language dominance: They both learn the L2 after the L1 and are less dominant in the L2. This makes disentangling the relative contribution of these two factors in translation priming difficult. To this end, the current experiment tested heritage Cantonese speakers in an auditory translation priming task. In heritage speakers, we observed marginally larger L2–L1 priming relative to L1–L2 priming, although heritage speakers showed priming in both directions. In native speakers, we only observed L1–L2 priming. Finally, English dominance positively correlated with L2–L1 priming and negatively correlated with L1–L2 priming. In sum, the current results suggest that both language dominance and order of acquisition are driving factors in the observed translation priming asymmetries, inviting a rethinking of lexical access in the context of bilingual speakers with various language dominance profiles.

## Supplemental Material

sj-pdf-1-qjp-10.1177_17470218221091753 – Supplemental material for Language dominance and order of acquisition affect auditory translation priming in heritage speakers Click here for additional data file. Supplemental material, sj-pdf-1-qjp-10.1177_17470218221091753 for Language dominance and order of acquisition affect auditory translation priming in heritage speakers by Rachel Soo and Philip J Monahan in Quarterly Journal of Experimental Psychology

sj-pdf-2-csv-10.1177_17470218221091753 – Supplemental material for Language dominance and order of acquisition affect auditory translation priming in heritage speakers Click here for additional data file. Supplemental material, sj-pdf-2-csv-10.1177_17470218221091753 for Language dominance and order of acquisition affect auditory translation priming in heritage speakers by Rachel Soo and Philip J Monahan in Quarterly Journal of Experimental Psychology
