# Supplementary material for: Language dominance and order of acquisition affect auditory translation priming in heritage speakers
Source: Q J Exp Psychol (Hove). 2022 May 7;76(2):284–93. doi: 10.1177/17470218221091753 (PMC9896266; doi:10.1177/17470218221091753)
Supplement: sj-pdf-1-qjp-10.1177_17470218221091753 – Supplemental material for Language dominance and order of acquisition affect auditory translation priming in heritage speakers [file sj-pdf-1-qjp-10.1177_17470218221091753.pdf]

Supplementary material for "Language dominance and order of acquisition affect auditory translation priming in heritage speakers".

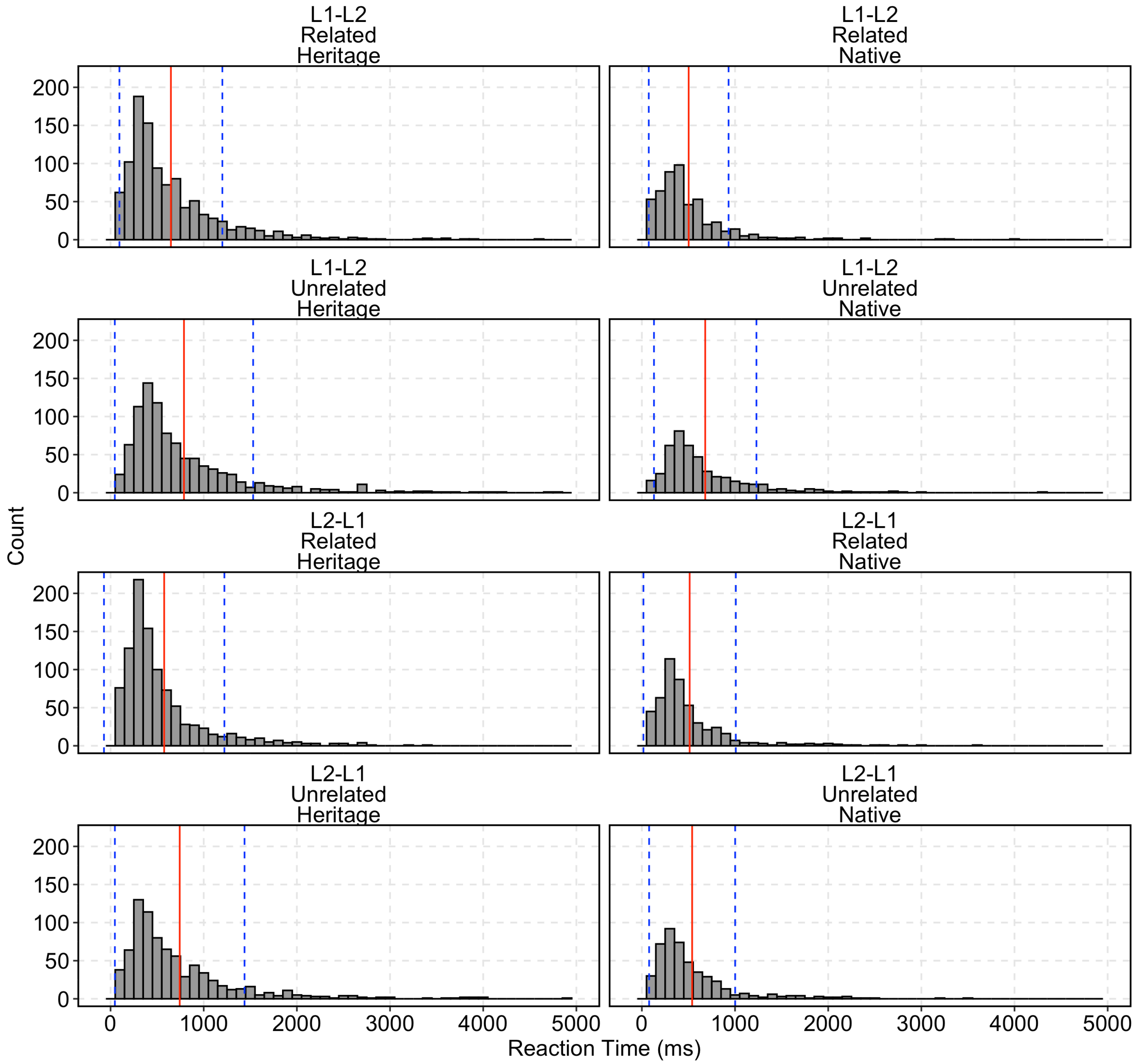

Figure A1. Reaction time distributions (ms) for heritage speakers (left) and native speakers (right) in each of the Direction and Relatedness conditions. The vertical solid red lines indicate the condition arithmetic mean, and the vertical blue dotted lines indicate one standard deviation from the mean.
